# P-869. Comparison of β-lactams Antibiotics Resistance of Viridans Streptococci in Bacteremic Children and Adults with Malignancy

**DOI:** 10.1093/ofid/ofae631.1060

**Published:** 2025-01-29

**Authors:** Rie Habuka, Yuta Aizawa, Masaru Imamura, Jun Takizawa, Akihiko Saitoh

**Affiliations:** Niigata University, Niigata, Niigata, Japan; Niigata University Graduate School of Medical and Dental Sciences, Niigata, Niigata, Japan; Department of Pediatrics, Niigata University Graduate School of Medical and Dental Sciences, Niigata, Niigata, Japan; Department of Hematology, Endocrinology and Metabolism, Niigata University Faculty of Medicine, Niigata, Niigata, Japan; Niigata University, Niigata, Niigata, Japan

## Abstract

**Background:**

Viridans streptococcus bacteremia (VSB) is an important complication after receiving chemotherapy in patients with malignancy. The increase in antibiotic resistance treating patients with VSB has been a major concern especially in children.

**Methods:**

This is a retrospective study to evaluate patients with malignancy diagnosed with VSB at Niigata University Hospital in Japan between January 2009 and May 2023. Clinical characteristics of the patients, and antibiotic susceptibility of viridans streptococci were evaluated. We used the Mann-Whitney test for numerical variables and the χ^2^ test, or Fisher’s exact test for categorical variables.

**Results:**

A total of 85 episodes (29 in children, 56 in adults) of VSB were identified during the study period. Acute myeloid leukemia was the most common diagnosis in both children (41%) and adults (61%). The susceptibility rate was significantly lower in children than adults; penicillin (45% vs. 79%, p =. 001), cefepime (52% vs. 91%, p < .001), and meropenem (55% vs. 89%, p < .001). Vancomycin was 100% susceptible in both groups. Based on the susceptibility results, the rate of appropriate initial antimicrobial coverage by empiric therapy was lower in children (59%) than in adults (91%) (p < .001). Severe complications included 2 meningitis cases in children, and a septic shock case in adults, 2 pediatric cases were treated with non-susceptible antibiotics at the onset of fever. No mortality was observed. The median number of previous febrile neutropenia episodes was higher in children (2, IQR 1-4) than adults (1, IQR 0-2) (p < .001). Additionally, the median duration of neutropenia at the onset was longer in children (6.5 days, IQR 4-11days) than adults (5 days, IQR 3-7days) (p = .03). No difference in β-lactam antibiotics use at the onset (7% vs. 0%, p = .11), or β-lactam exposure within 30 days of onset (41% vs. 41%, p = .98) was observed between child and adults.

**Conclusion:**

We demonstrated a high rate of β-lactam antibiotics resistance to viridans streptococci in patients with malignancy, especially in children. The frequent antimicrobial exposure in children might be related to such high resistance. Vancomycin may need to be included in an empiric therapy in the setting where β-lactam antibiotics resistance of viridans streptococci is high.

**Disclosures:**

**All Authors**: No reported disclosures

